# An acid-responsive bone-targeting nanoplatform loaded with curcumin balances osteogenic and osteoclastic functions

**DOI:** 10.1093/rb/rbaf028

**Published:** 2025-05-05

**Authors:** Minhao Liang, Lei Zhou, Juan Li, Bin Liang, Liangyun Zhou, Fengfeng Xue, Libo Jiang, Wei Hong

**Affiliations:** Shanghai Institute of Geriatrics and Gerontology, Huadong Hospital, Fudan University, Shanghai 200040, China; Shanghai Key Laboratory of Clinical Geriatric Medicine, Huadong Hospital, Fudan University, Shanghai 200040, China; Department of Orthopedic Surgery, Zhongshan Hospital, Fudan University, Shanghai 200032, China; Department of Orthopaedic Surgery, Shanghai Geriatric Medical Center, Shanghai 201104, China; Department of Orthopedic Surgery, Zhongshan Hospital, Fudan University, Shanghai 200032, China; Shanghai Institute of Geriatrics and Gerontology, Huadong Hospital, Fudan University, Shanghai 200040, China; Shanghai Key Laboratory of Clinical Geriatric Medicine, Huadong Hospital, Fudan University, Shanghai 200040, China; Department of Functional Intestinal Diseases, General Surgery of Shanghai Tenth People’s Hospital, Tongji University School of Medicine, Shanghai 200070, China; Department of Orthopedic Surgery, Zhongshan Hospital, Fudan University, Shanghai 200032, China; Department of Orthopedic Surgery, Zhongshan Hospital (Xiamen Branch), Fudan University, Shanghai 200032, China; Shanghai Institute of Geriatrics and Gerontology, Huadong Hospital, Fudan University, Shanghai 200040, China; Shanghai Key Laboratory of Clinical Geriatric Medicine, Huadong Hospital, Fudan University, Shanghai 200040, China; National Clinical Research Center for Aging and Medicine, Huashan Hospital, Fudan University, Shanghai 200040, China

**Keywords:** osteoporosis, curcumin, nanoparticle, bone-targeting, bone homeostasis

## Abstract

Postmenopausal osteoporosis (PMOP) is a predominant form of clinical osteoporosis. It has led to significant health and social burdens for older patients. Reestablishing the balance between osteogenic and osteoclastic is a crucial strategy for treating PMOP. Curcumin (Cur), a naturally derived polyphenolic substance, has gained recognition as a viable option for treating osteoporosis. Despite its potential, the clinical use of Cur is hindered by its limited bioavailability and the presence of side effects. Nanoparticles modified with aspartic acid octapeptide (ASP8) exhibit a strong affinity for bone tissue, facilitating targeted delivery. This study presents novel acid-responsive zeolite imidazolate framework-8 (ZIF) nanoparticles modified with ASP8 and loaded with Cur (Cur@ZIF@ASP8, CZA). Upon delivery by this nanoparticle drug delivery system, Cur can effectively regulate bone homeostasis, offering a potential therapeutic strategy for osteoporosis. This study demonstrated that CZA nanoparticles could successfully transport Cur to bone tissue without significant toxicity. Furthermore, nanoparticles promote bone formation and inhibit osteoclast activity. They also modify the expression of related genes and proteins, such as OCN, ALP, CTSK and MMP9. Significant evaluations utilizing microcomputed tomography, Masson’s staining, hematoxylin and eosin staining and immunofluorescence staining demonstrated that intravenous CZA administration in ovariectomized mice resulted in bone destruction while simultaneously reducing overall bone loss. In conclusion, CZA nanoparticles hold promise as a therapeutic option for osteoporosis.

## Graphical abstract

**Figure:**
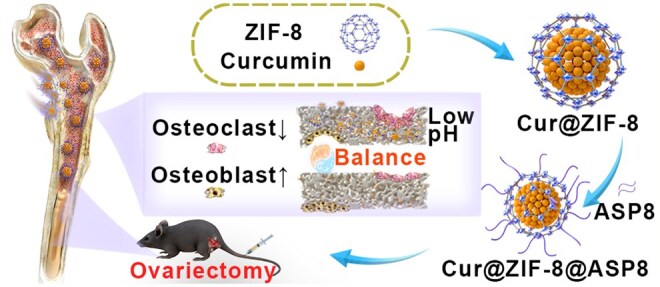

## Introduction

Postmenopausal osteoporosis (PMOP) is the most common type of osteoporosis, characterized by a decrease in bone mineral density (BMD) and a heightened risk of fractures. As the population worldwide continues to age, PMOP has become a major public health issue on an international level [1].

Bone homeostasis represents a physiological mechanism that is modulated by the interplay of osteoblasts and osteoclasts. This process is inherently marked by the synchronized functions of these two cell types. A key determinant in PMOP development is the disruption between bone formation and resorption by osteoblasts and osteoclasts, respectively [2]. At present, the main clinical approaches for treating PMOP involve pharmacological strategies aimed at modulating bone homeostasis by either suppressing bone resorption or enhancing bone formation. Therapeutic approaches include hormone replacement therapy and the use of medications such as bisphosphonates, denosumab, selective estrogen receptor modulators, calcitonin, parathyroid hormone and strontium salts [1]. However, these pharmacological alternatives exhibit several common limitations, such as limited specificity in targeting delivery to the bone, potential adverse effects on other organs and insufficient circulation time. Moreover, the complex underlying mechanisms of osteoporosis frequently involve multiple targets, suggesting that therapies focused solely on osteoblasts or osteoclasts are insufficient to reestablish homeostasis within the osteoporotic microenvironment. Therefore, the development of innovative and more efficacious treatment approaches is essential for enhancing the quality of life in patients suffering from PMOP.

Curcumin (Cur), a natural polyphenol extracted from turmeric roots, exhibits a range of therapeutic properties, including anti-inflammatory, antioxidant, hypoglycemic and anticancer activities [3]. Cur has been utilized to treat diseases, such as diabetes, osteoarthritis and Alzheimer’s disease [4]. Recent research suggests that Cur may aid in osteoporosis prevention and treatment by inhibiting bone resorption while promoting bone formation [5]. In addition, Cur has disadvantages, such as autoxidation, inadequate water solubility and suboptimal targeting, which consequently diminished bioavailability and limited its clinical applicability [6].

Nanomaterials offer several advantages, such as reduced drug side effects, optimal drug stability and sustained and controlled drug release, making nanomaterials an ideal choice for drug delivery systems [7, 8]. Among nanoparticles, those based on metal–organic frameworks (MOFs) address various clinical challenges. MOFs have numerous characteristics, such as adjustable pore sizes, flexible structures and high drug-loading capacities [9]. In recent years, a novel metal–organic compound composed of Zn^2+^ and 2-methylimidazole, called zeolite imidazolate framework-8 (ZIF), has become a significant research focus in cancer treatment, bioimaging and bioantibacterial applications [10, 11]. Beyond the typical benefits associated with MOFs, ZIF exhibits notable biocompatibility and the capability to degrade under acidic conditions. Consequently, ZIF serves as a proficient carrier for proteins and enzymes, effectively maintaining their biological activity while simultaneously improving their functional performance and stability. Motivated by the unique binding characteristics of noncollagenous proteins present in the bone matrix, including osteopontin (OPN) and osteocalcin (OCN), researchers have investigated the use of acidic oligopeptides made up of aspartic or glutamic acid. These oligopeptides are being explored as ligands for delivering drugs specifically to bone tissues [12]. Aspartic acid octapeptide (ASP8) can improve nanomaterial delivery to bone tissue through calcium chelation on bone mineral surfaces. To modify nanodrug carriers for the treatment of malignant tumor bone metastasis and osteoporosis, recent studies have reported the utilization of ASP8 as a ligand that can target active sites of bone resorption [13, 14].

Herein, Cur@ZIF@ASP8 (CZA), a novel Cur-loaded nanoparticle that utilizes ASP8, was designed to impart targeting capabilities and enhance nanoparticle accumulation in the bone microenvironment. Moreover, we took advantage of ZIF’s ability to break down in acidic environments to release Cur. This controlled release promoted the differentiation of osteogenic cells and supported the formation of new bone tissue. We hypothesize that these nanoparticles can enhance the bone remodeling process and hold promise for advancing osteoporosis treatment.

## Materials and methods

### Chemicals

Methanol (M433273), Zn(NO_3_)_2_·6H_2_O, 2-methylimidazole (2-MI), Cur (C463317) and dimethyl sulfoxide (D103277) were purchased from Aladdin Chemicals.

### Characterization

A JEM-2100F microscope operating at an accelerating voltage of 200 kV was employed to perform the transmission electron microscopy (TEM) analysis. The assessment of hydrodynamic diameters and zeta potentials was conducted using a Zetasizer Nano series device (Nano ZS90). A D/Max-2550 V diffractometer (Rigaku, Japan) was employed to obtain the powder X-ray diffraction pattern, equipped with a CuKα radiation source set at 40 kV and 40 mA. Ultraviolet–visible (UV–vis) spectra were obtained using Shimadzu’s UV-3600 spectrophotometer from Kyoto, Japan. Fluorescence measurements were performed with the RF-5301 PC spectrophotometer, also by Shimadzu. Additionally, nitrogen adsorption and desorption isotherms were analysed utilizing the BELSORP MaxII analyser.

### Synthesis of ZIF and Cur@ZIF

A solution of 2-MI (480 mg) was prepared in 30 ml of methanol, with or without the addition of Cur (15.0 mg). Subsequently, 30 ml of deionized water containing Zn(NO_3_)_2_·6H_2_O (450 mg) was gradually introduced into the mixtures over a period of 10 min under magnetic stirring, followed by an additional 10 min of mixing. The resulting solutions were then stirred continuously for 30 min. The nanoparticles were purified by centrifugation at 10 000 rpm and rinsed with ethanol to remove impurities.

### Synthesis of Cur@ZIF@ASP8

Cur@ZIF (CZ) (15 mg) was initially suspended in 30 ml of ethanol. Subsequently, a solution of deionized water containing 1.5 mg of ASP8 was incrementally introduced to the ethanol solution over a duration of 10 min while maintaining magnetic stirring. Subsequently, the mixture was continuously stirred for additional 10 min. Later, stirring was extended by 6 h. Centrifugation at 13 000 rpm for 10 min was employed to isolate and purify the resulting particles.

### Drug release analysis

To establish a standard curve for Cur, the average absorbance values of solutions containing different concentrations of Cur were recorded. To determine the Cur content in CZA, 10 mg of CZA was entirely dissolved in 40 ml of phosphate-buffered saline (PBS) adjusted to pH values of 5.5, 6.5 and 7.5. Subsequently, each resulting solution’s absorbance was measured at 420 nm using a UV–vis spectrophotometer. The absorbance of each prepared solution was measured at a wavelength of 420 nm using a UV–vis spectrophotometer. The concentration of Cur in the unidentified sample was determined by interpolating its absorbance value against the constructed standard curve.

### Cell culture

MC3T3-E1 and RAW264.7 cell lines were sourced from the Cell Bank of the Chinese Academy of Sciences (Shanghai, China). These cells were maintained in alpha-minimum essential medium (α-MEM) supplemented with 10% fetal bovine serum and incubated under 5% CO_2_ at 37°C within a humidified environment.

### *In vitro* biocompatibility assay

MC3T3-E1 and RAW264.7 cells were seeded into 96-well plates, and 10 µl of either PBS or a PBS solution containing one of the four drugs (ZIF, Cur, CZ or CZA) at concentrations of 2.5, 5, 10, 20, 40 or 80 ng/ml was added to each well. Triplicate wells were used for each condition. After 1, 2 or 3 days, Cell Counting Kit-8 (CCK-8) solution (Solarbio, China) was introduced into each well and incubated for 2 h. The optical density (OD) was measured at 462 nm, and the results were presented as means ± standard deviations (SDs). Furthermore, after 3 days of incubation, cell survival was assessed using a calcein-AM/propidium iodide double-staining kit (Beyotime, China), following the same experimental protocol.

### Cellular uptake of the nanoparticles

Fluorescein isothiocyanate (FITC) was used to label the nanoparticles. MC3T3-E1 cells were seeded in 24-well plates and incubated with the nanoparticles for 0.5, 1 or 2 h. After the incubation, the cells were stained with 4′,6-diamidino-2-phenylindole (DAPI) and phalloidin. The samples were then analysed using confocal microscopy to assess the results.

### Osteogenic activity of the nanoparticles *in vitro*

An osteogenic induction medium comprising 10 mM sodium β-glycerophosphate, 100 μM sodium ascorbate and 10 μM dexamethasone was used to culture MC3T3-E1 cells. PBS or a PBS solution containing one of three drugs (ZIF, Cur or CZ at 5 mg/ml) was added to the medium, which was replaced every 2 days. A BCIP/NBT kit (MA0197, Meilunbio) was used for the qualitative analysis of osteoblast activity on days 7, 14 and 21, while Alizarin Red S (ARS) staining was used on the same day to evaluate osteoblast mineralization. On day 7, mRNA and protein were extracted for further analysis through reverse-transcription quantitative polymerase chain reaction (RT-qPCR) and Western blotting.

### Osteoclast-inhibiting ability of the nanoparticles *in vitro*

RAW264.7 cells were cultured in an osteoclast induction medium supplemented with 50 ng/ml receptor activator of nuclear factor kappa beta (RANKL) and either PBS alone or PBS containing one of three drugs (ZIF, Cur or CZ at a concentration of 5 mg/ml). The medium was replaced every two days. On the seventh day of induction, tartrate-resistant acid phosphatase (TRAP) staining was performed to assess osteoclast activity, while mRNA and protein were simultaneously extracted for subsequent qPCR and Western blot analyses.

### RT-qPCR analysis of the cultured cells

Total RNA was isolated from cells using TRIzol reagent (15596026, Gibco, USA), and complementary DNA (cDNA) was synthesized using the PrimeScript^™^ RT Master Mix (Perfect Real Time, RR036A, Takara). Quantitative reverse-transcription polymerase chain reaction (RT-qPCR) was conducted on the Applied Biosystems StepOne system. Gene expression levels were quantified using the 2^−ΔΔ^^*Ct*^ method, with GAPDH serving as the internal control. The primer sequences used in this analysis are listed in Table 1.

**Table 1.** Sequences of primers used for RT-qPCR in this study

Primer	Forward (5′–3′)	Reverse (5′–3′)
GAPDH	AGGTCGGTGTGAACGGATTTG	TGTAGACCATGTAGTTGAGGTCA
OCN	GCAGCTTGGTGCACACCTAG	GGAGCTGCTGTGACATCCAT
RUNX2	CCACAAGGACAGAGTCAGAT	GATAGGAGGGGTAAGACTGG
COL1A1	GCTCCTCTTAGGGGCCACT	CCACGTCTCACCATTGGGG
MMP9	CTGGACAGCCAGACACTAAAG	CTCGCGGCAAGTCTTCAGAG
CTSK	GAAGAAGACTCACCAGAAGCAG	TCCAGGTTATGGGCAGAGATT
TRAP	CAGCAGCCAAGGAGGACTAC	ACATAGCCCACACCGTTCTC

### Western blotting

After cell culture, proteins were extracted via electrophoresis and transferred onto polyvinylidene fluoride (PVDF) membranes (IPFL00010, Sigma-Aldrich, MO, USA). The membranes were blocked for 1 h at room temperature with a 5% bovine serum albumin solution (Sigma-Aldrich) and then incubated overnight at 4°C with primary antibodies (Affinity, SF, USA) diluted 1:1000. This was followed by a 1-h room temperature incubation with secondary antibodies diluted 1:10 000. Protein detection was conducted using the Pierce ECL Western blotting substrate (Biosharp, China). Proteins of interest were isolated seven days after nanoparticle exposure, and their quantification was performed using ImageJ.

### Labeling CZA with IR780

Briefly, 1 mg of the fluorescent dye IR780, solubilized in 400 µl of dimethyl sulfoxide, was incorporated into a CZA solution (20 mg dissolved in 20 ml of deionized water) within a small flask designed to block light. This mixture underwent stirring at 26°C for 24 h. Subsequently, any unbound dye was eliminated through centrifugation at a speed of 10 000 rpm, and the resultant product was subsequently washed with water on three separate occasions.

### Targeting of the nanoparticles *in vivo*

Eight-week-old female C57/BL6J mice (15–20 g) were administered 200 μl of either PBS or IR780-labeled CZ or CZA nanoparticles via tail vein injection. The nanoparticles, prepared in saline at a dose of 5 mg/kg, were tracked at 4, 8 and 12 h postinjection. Their biodistribution was assessed using an *in vivo* fluorescence imaging system (IVIS Spectrum, PerkinElmer, MA, USA). (Ex [excitation wavelength] = 780 nm, Em [emission wavelength] = 800 nm, time = 10 s).

### Biocompatibility of the nanoparticles *in vivo*

C57/BL6J mice received tail vein injections of 200 μl of PBS, ZIF, CZ or CZA every 3 days. The nanoparticles were prepared in saline at a concentration of 5 mg/kg. After four injections, the mice were euthanized, and blood and serum samples were collected. Whole blood samples were analysed to determine red blood cell, white blood cell and platelet counts, as well as hemoglobin and hematocrit levels. Serum samples were used to measure creatinine, alanine aminotransferase, aspartate aminotransferase and urea nitrogen levels. The tissues from the heart, liver, spleen and kidneys were harvested and subjected to hematoxylin and eosin (HE) staining for histological evaluation.

### Therapeutic potential of the nanoparticles *in vivo*

The experimental animals were allocated into the ZIF, CZ, CZA, OVX (blank) and negative normal (sham) groups via the random number table method. To simulate human PMOP, a mouse model of osteoporosis was established by performing ovariectomy. Anesthesia was achieved via intraperitoneal administration of sodium pentobarbital at a dosage of 30 mg/kg, after which both ovaries were entirely excised. The incision was closed by suturing the muscle layer and skin, and the surgical site was carefully disinfected. In the negative control group, the ovaries were left untouched, with only a small section of the surrounding adipose tissue being removed. Various nanoparticles were suspended in normal saline to prepare the required solutions.

After successfully establishing the OVX mouse model, the mice were administered injections of the corresponding nanoparticles every 3 days for a duration of 4 weeks. Upon conclusion of the treatment, the animals were euthanized, and both femoral and serum samples were collected. Femoral samples underwent initial analysis via microcomputed tomography (micro-CT) and were then preserved in 4% paraformaldehyde for subsequent investigations. Once satisfactory fixation was achieved, the samples were decalcified with ethylenediaminetetraacetic acid, embedded in paraffin to create wax blocks and sectioned. Finally, the bone sections were subjected to Masson’s staining, HE staining, TRAP staining and immunofluorescence detection. To assess indicators associated with osteogenesis and osteoclast activity, mouse serum samples were subjected to enzyme-linked immunosorbent assays (ELISAs) in accordance with the protocols provided by the manufacturer.

Animal experiments were conducted with approval from the Institutional Animal Care and Use Committee of Fudan University (2024-057) and adhered to the Laboratory Animal Care and Use Guidelines established by the National Institutes of Health.

### Statistical analysis

The results are presented as means ± SD based on at least three independent experiments. Statistical analyses were performed using two-sided *t*-tests and one-way ANOVA to evaluate significance. Kaplan–Meier survival curves were analysed using the log-rank Mantel–Cox test for comparison. A *P* value of <0.05 was regarded as statistically significant. All statistical computations were carried out using GraphPad Prism 8 (GraphPad Software Inc., La Jolla, CA).

## Results

### CZA synthesis and characterization

**Figure 1.**
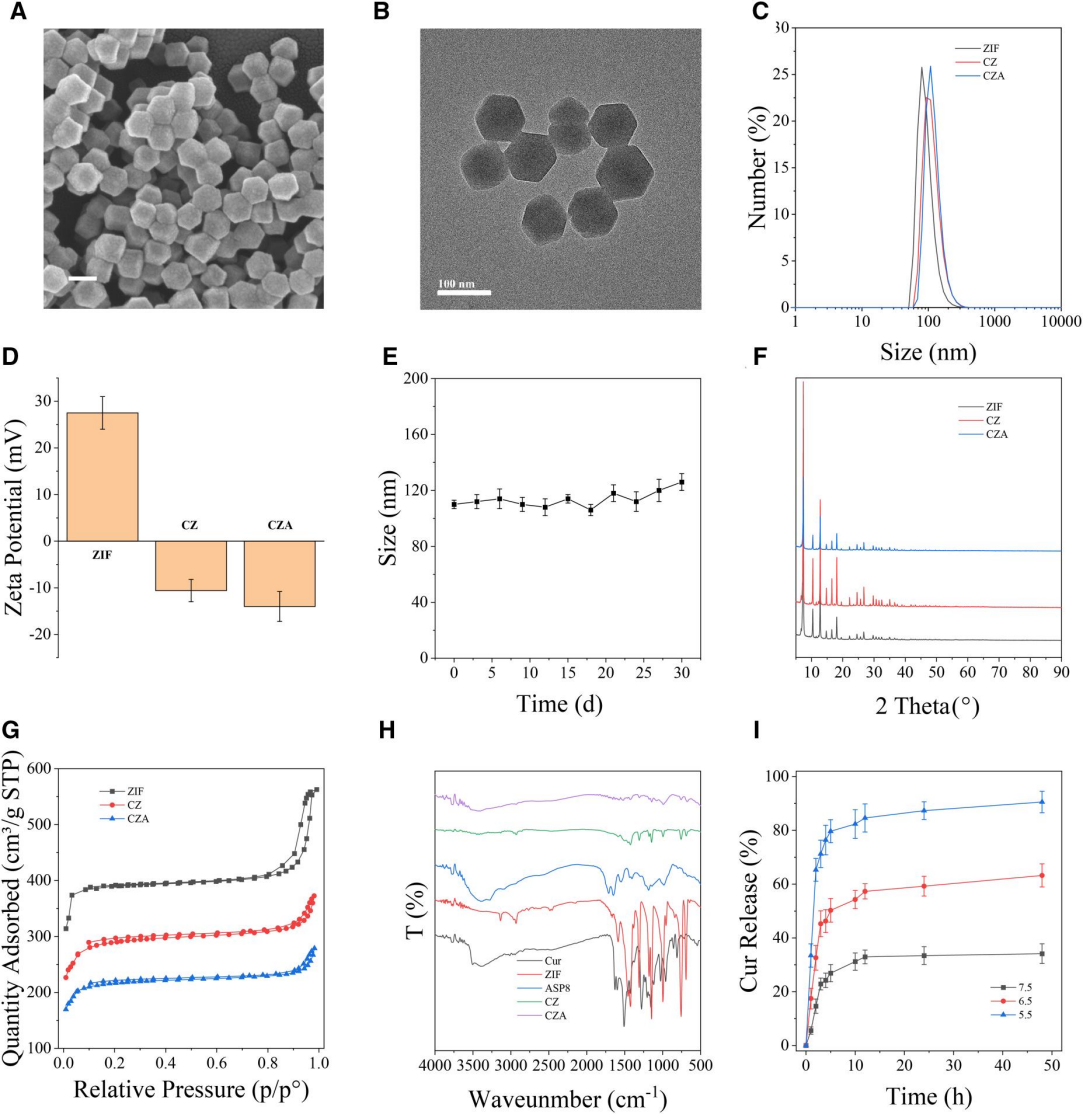
Characterization of the CZA nanoparticles. (**A**) SEM image of CZA. (**B**) TEM image of CZA. (**C**) DLS analysis comparing particle size distributions of ZIF, CZ and CZA in deionized water. (**D**) Zeta potential measurements of ZIF, CZ and CZA in deionized water. (**E**) Stability assessment of CZA hydrodynamic diameters after 30 days in PBS. (**F**) XRD patterns of ZIF, CZ and CZA, highlighting crystallographic structures. (**G**) BET surface area analysis for ZIF, CZ and CZA. (**H**) FTIR spectra of ASP8, Cur, ZIF, CZ and CZA, identifying characteristic functional groups. (**I**) Cur release profile from CZA in PBS with pH values of 5.5, 6.5 and 7.5 over 48 h *in vitro*. BET, Brunauer–Emmett–Teller; Cur, curcumin; CZ, Cur@ZIF; CZA, Cur@ZIF@ASP8; DLS, dynamic light scattering; FTIR, Fourier transform infrared spectroscopy; PBS, phosphate-buffered saline; XRD, X-ray diffraction; ZIF, zeolite imidazolate framework-8.

The scanning electron microscopy (SEM) image of CZA is shown in Figure 1A, which reveals that CZA is monodisperse with a hexagonal morphology. The TEM image of CZA (Figure 1B) confirmed the hexagonal morphology of CZA with a diameter of 94 ± 5 nm. Supplementary Figure S1 presents SEM and TEM images, revealing that ZIF and Cur@ZIF exhibit comparable morphologies without any significant differences. Figure 1C displays the results of dynamic light scattering measurements, indicating that the hydrodynamic diameters for ZIF, CZ and CZA are 78, 100 and 108 nm, respectively. Figure 1D illustrates that the zeta potentials of ZIF, CZ and CZA were measured to be 27.5 ± 3.5, −10.6 ± 2.4 and −14 ± 3.2 mV, respectively. After loading Cur and ASP8, the hydrodynamic diameter of CZA increased compared with those of ZIF and CZ, and the zeta potential of CZA changed from positive to negative, indicating that Cur and ASP8 had been loaded into ZIF. CZA demonstrated good dispersion in PBS, with no precipitation detected even after being stored at 4°C for over one month (Figure 1E). X-ray diffraction analysis of CZA (Figure 1F) revealed that the crystal structure of the prepared ZIF-8 was consistent with previous results, and the structural integrity remained largely unchanged after and ASP8 loadings. The Brunauer–Emmett–Teller (BET) surface areas of ZIF, CZ and CZA were measured as 1683.1, 1149.3 and 876.0 m^2^/g, respectively (Figure 1G). In addition, Fourier transform infrared spectroscopy analysis (Figure 1H) validated the incorporation of Cur and ASP8 into CZA. The Cur-loaded CZA was calculated from the UV absorption spectrum and was determined to be 67.8 μmol/g. ZIF is a typical pH-responsive drug release carrier with excellent pH-responsive drug release performance. Figure 1I presents the evaluation of Cur release from CZA in environments with various pH levels. The analysis revealed that Cur is liberated from CZA at a pH of 5.5. In contrast, the carrier demonstrates increased stability when the pH is adjusted to 7.4. This indicates that CZA maintains its responsiveness to an acidic microenvironment.

### Biocompatibility and cellular uptake of the nanoparticles *in vitro*

**Figure 2.**
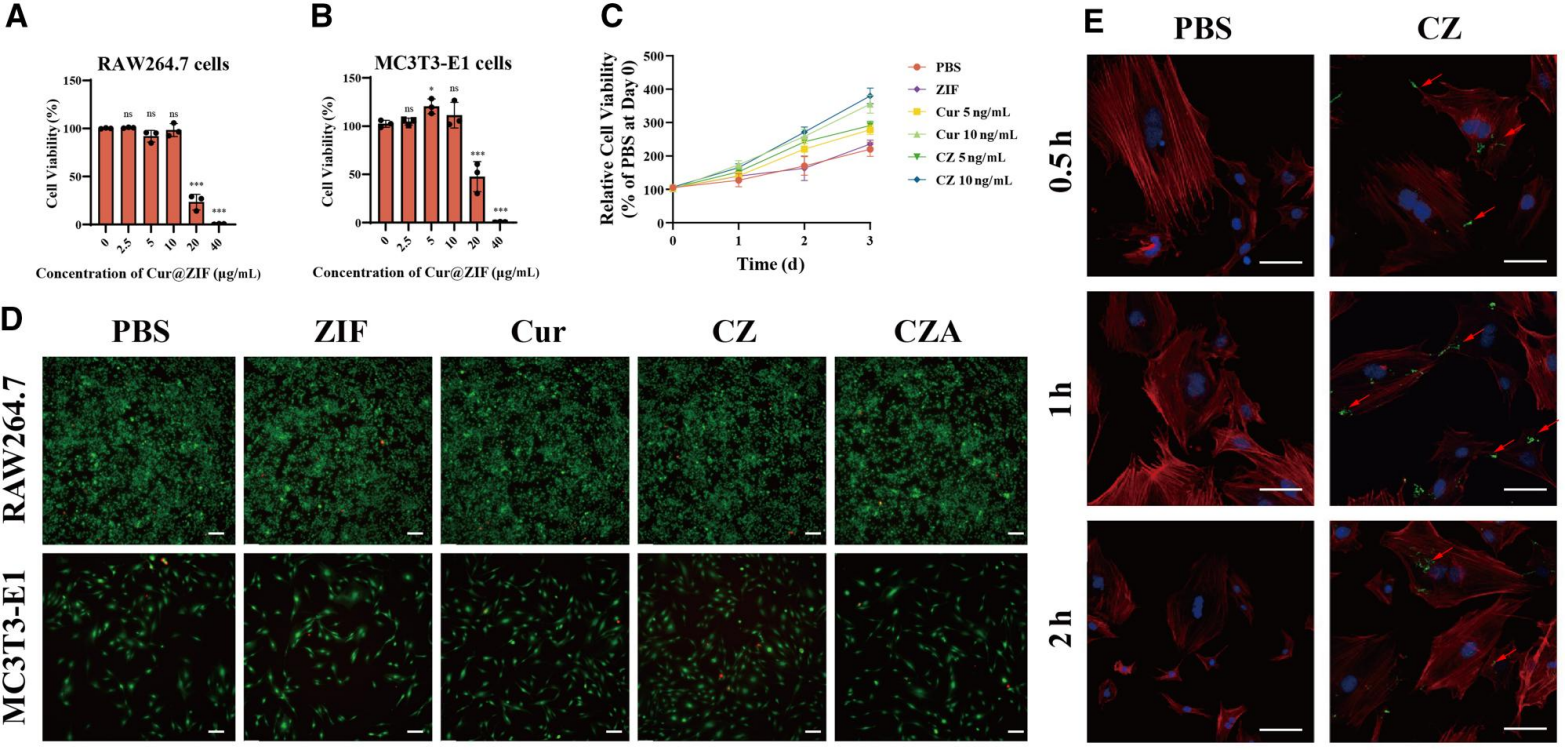
Biocompatibility and cellular uptake of the nanoparticles *in vitro*. (**A**) Cell viability of RAW264.7 cells cultured with varying concentrations of CZ for 3 days, measured via the CCK-8 assay (*n* = 3, compared to 0 μg/ml). (**B**) Cell viability of MC3T3-E1 cells cultured with different concentrations of CZ on day 3, as determined by the CCK-8 assay (*n* = 3, compared with 0 μg/ml). (**C**) Proliferation of MC3T3-E1 cells over time, evaluated through the CCK-8 assay. (**D**) Live/dead staining images of cells following 3 days of incubation (scale bar = 100 μm). (**E**) Visualization of *in vitro* cellular uptake of FITC-labeled CZ by MC3T3-E1 cells, as shown by fluorescence microscopy (scale bar = 20 μm). (ns: *P* > 0.05, *P < 0.05, **P < 0.01, ***P < 0.001). CCK-8, Cell Counting Kit-8; Cur, curcumin; CZ, Cur@ZIF; CZA, Cur@ZIF@ASP8; DAPI, 4′,6-diamidino-2-phenylindole; FITC, fluorescein isothiocyanate; PBS, phosphate-buffered saline; ZIF, zeolite imidazolate framework-8.

Nanoparticles must be biocompatible for their utilization. Figure 2A and B shows the survival rates of RAW264.7 and MC3T3-E1 cells in various groups with different compound concentrations. The findings demonstrated that CZA exhibited no notable toxicity toward RAW264.7 or MC3T3-E1 cells at concentrations below 10 µg/ml. Interestingly, when nanoparticles were incubated with MC3T3-E1 cells for 1, 2 or 3 days, the OD values for both the Cur and nanoparticle-treated groups exceeded those of the control group, displaying a clear concentration-dependent trend (Figure 2C). These findings show that Cur is nontoxic and can effectively promote MC3T3-E1 cell proliferation in a dose-dependent manner. Living and dead cells were stained following a 3-day coculture period. All groups of MC3T3-E1 cells exhibited strong green fluorescence, which indicates live cells, with few dead cells, which were indicated by red fluorescence (Figure 2D). To investigate cellular uptake of the nanoparticles, CZA nanoparticles were fluorescently labeled with FITC, while the cells were stained using rhodamine-conjugated phalloidin. Confocal microscopy observations after 0.5, 1 and 2 h of coculture revealed that the cells exhibiting green fluorescence had internalized the nanoparticles, indicating successful uptake (Figure 2E). This finding also indicates the absence of short-term cytotoxicity and confirms the effective interaction between nanoparticles and cells. This cellular endocytosis of the nanoparticles further shows their favorable biocompatibility and cellular affinity. As a result, CZA demonstrates potential as a therapeutic agent for modulating bone remodeling processes.

### Osteogenic activity and osteoclast-inhibiting ability of the nanoparticles *in vitro*

**Figure 3.**
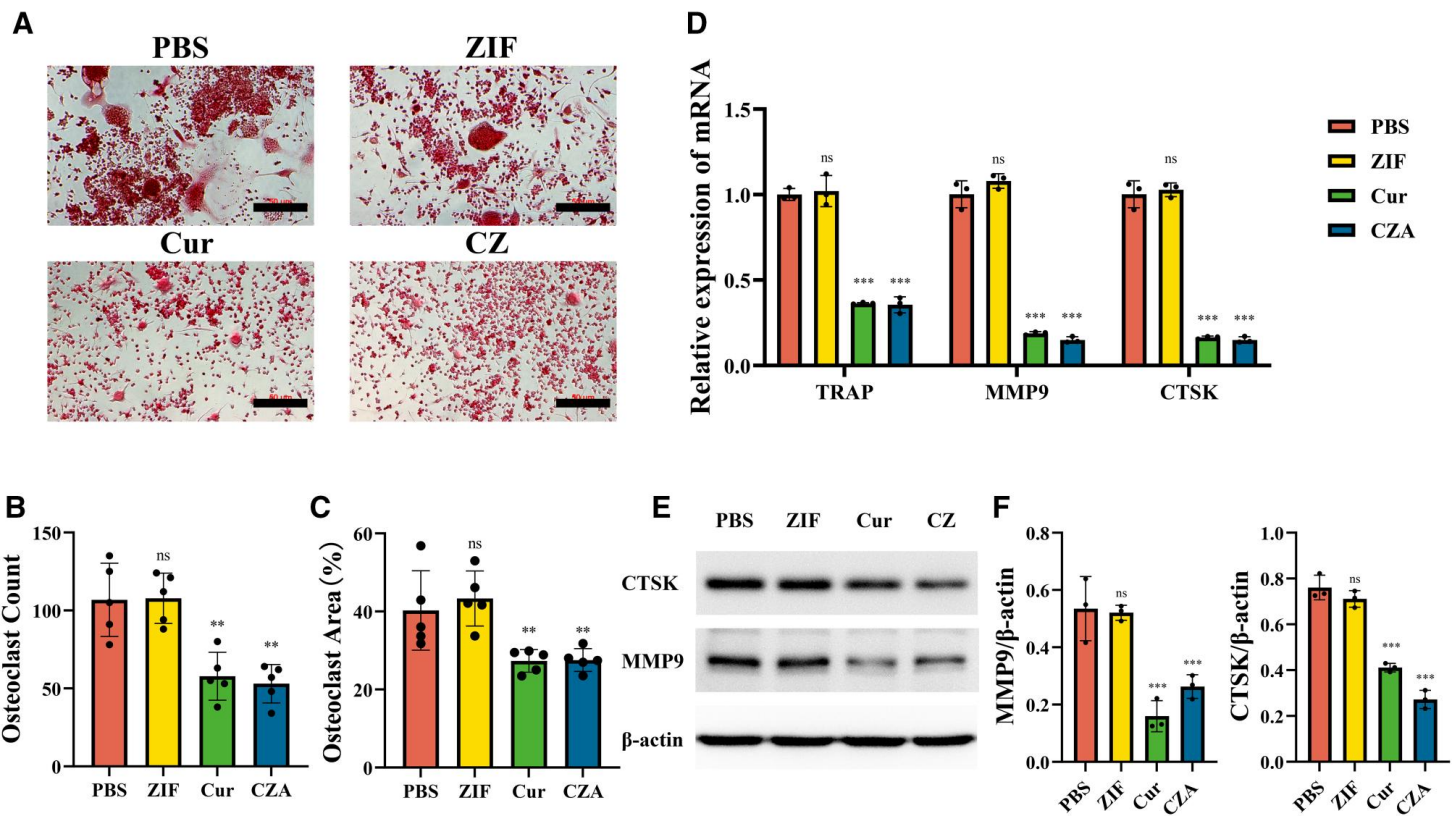
Effects of the nanoparticles on the osteoclast differentiation of RAW264.7 cells. (**A**) TRAP staining of RAW264.7 cells in various groups after 7 days of RANKL-induced osteoclast differentiation. (**B**) Osteoclast counts in different groups after induction (*n* = 5, compared with the PBS group). (**C**) Osteoclast area in different groups after induction (*n* = 5, compared with the PBS group). (**D**) Relative mRNA expression of osteogenesis-related genes (TRAP, MMP9 and CTSK) in the PBS, ZIF, Cur and CZ groups after induction (*n* = 3). (**E**) Western blotting analysis of MMP9 and CTSK expression. (**F**) Semiquantitative analysis of Western blotting (ns: *P* > 0.05, *P < 0.05, **P < 0.01, ***P < 0.001). Cur, curcumin; CZ, Cur@ZIF; PBS, phosphate-buffered saline; RANKL, receptor activator of nuclear factor kappa beta; TRAP, tartrate-resistant acid phosphatase; ZIF, zeolite imidazolate framework-8.

The regulation of osteogenesis and osteoclast function is a key mechanism by which nanoparticles can be used to treat PMOP [15]. A previous study demonstrated that Cur inhibited osteoclast differentiation while promoting osteogenesis [16]. Initially, whether these Cur nanoparticles could inhibit osteoclast function *in vitro* was verified in the present study. RAW264.7 cells undergo differentiation into multinucleated osteoclasts upon stimulation with RANKL. To assess the ability of Cur-loaded nanoparticles to inhibit osteoclast differentiation, the nanoparticles were introduced to RAW264.7 cells during the process of RANKL-induced osteoclastic differentiation. The cultures were then examined on the seventh day of differentiation to assess their effects. TRAP staining indicated that the Cur nanoparticles effectively inhibited the formation of multinucleated osteoclasts (Figure 3A–C). MMP9 and CTSK have been established as osteoclast marker proteins [17–19]. The RT-qPCR analysis revealed a notable decrease in MMP9 and CTSK mRNA expression in the CZ and CZA groups when compared to the blank and blank ZIF groups (Figure 3D). Correspondingly, Western blot analysis demonstrated reduced protein expression levels of these markers, which were consistent with the trends observed in mRNA expression (Figure 3E and F). These findings suggest that the Cur nanoparticles effectively suppress osteoclast differentiation.

**Figure 4.**
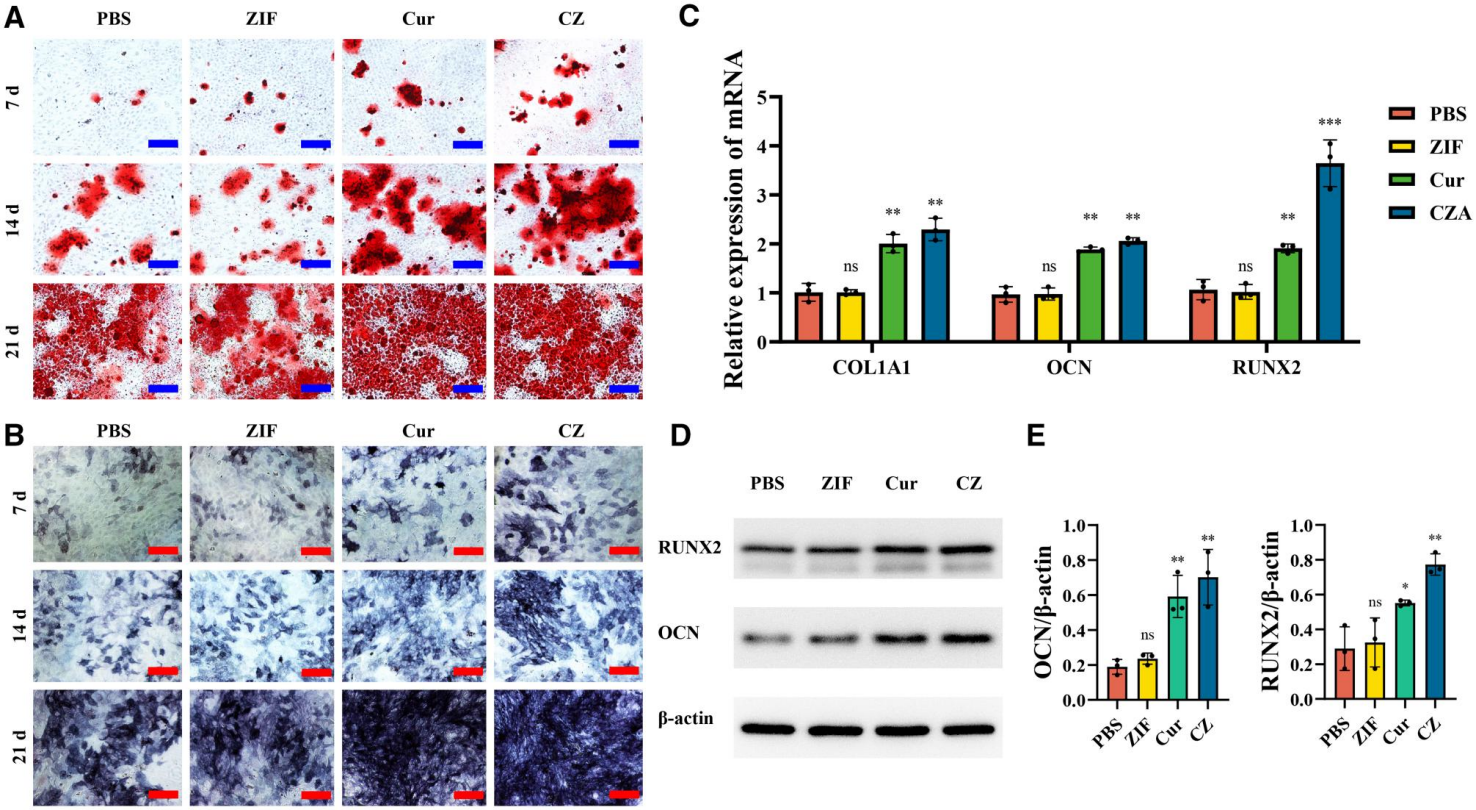
Nanoparticles promoted osteogenesis *in vitro*. (**A**) Images of ARS staining showing MC3T3-E1 cells across different groups at days 7, 14 and 21. (**B**) Images of ALP staining showing MC3T3-E1 cells across different groups on days 7, 14 and 21. (**C**) Quantitative analysis of relative mRNA levels for osteogenesis-related genes in MC3T3-E1 cells from different groups on day 7 following osteogenic induction (*n* = 3). (**D**) Western blotting analysis of RUNX2 and ALP expression. (**E**) Semiquantitative analysis of Western blotting (ns: *P* > 0.05, *P < 0.05, **P < 0.01, ***P < 0.001). ALP, alkaline phosphatase; ARS, Alizarin Red S; Cur, curcumin; CZ, Cur@ZIF; PBS, phosphate-buffered saline; ZIF, zeolite imidazolate framework-8.

The impact of Cur nanoparticles on osteogenic activity was further evaluated. Alkaline phosphatase (ALP) secretion and calcium salt deposition, which are key indicators of osteogenic differentiation, were analysed. ALP and ARS staining provided distinct evidence supporting osteogenic differentiation in MC3T3-E1 cells. ALP and ARS staining progressively increase, indicating time-dependent enhancement over the course of 7, 14 and 21 days of differentiation (Figure 4A and B). Notably, the Cur and CZ groups exhibited elevated ALP expression and increased calcium salt deposition when compared with the blank control and ZIF groups. Furthermore, the impact of Cur nanoparticles on the expression of osteogenesis-associated factors in MC3T3-E1 cells was assessed. Results from RT-qPCR demonstrated that Cur nanoparticles enhanced the mRNA expression of bone-related genes, including COL1A1, OCN and RUNX2 (Figure 4C) as well as elevated the protein levels of ALP and RunX2 (Figure 4D and E).

These findings implied that the functions of the Cur nanoparticles are similar to those of Cur at equivalent concentrations *in vitro*. Consequently, further in-depth studies and discussion are necessary to elucidate how Cur regulates osteogenic function and identify the mechanisms underlying the osteoclast signaling pathways.

### Bone-targeting ability of the nanoparticles *in vivo*

**Figure 5.**
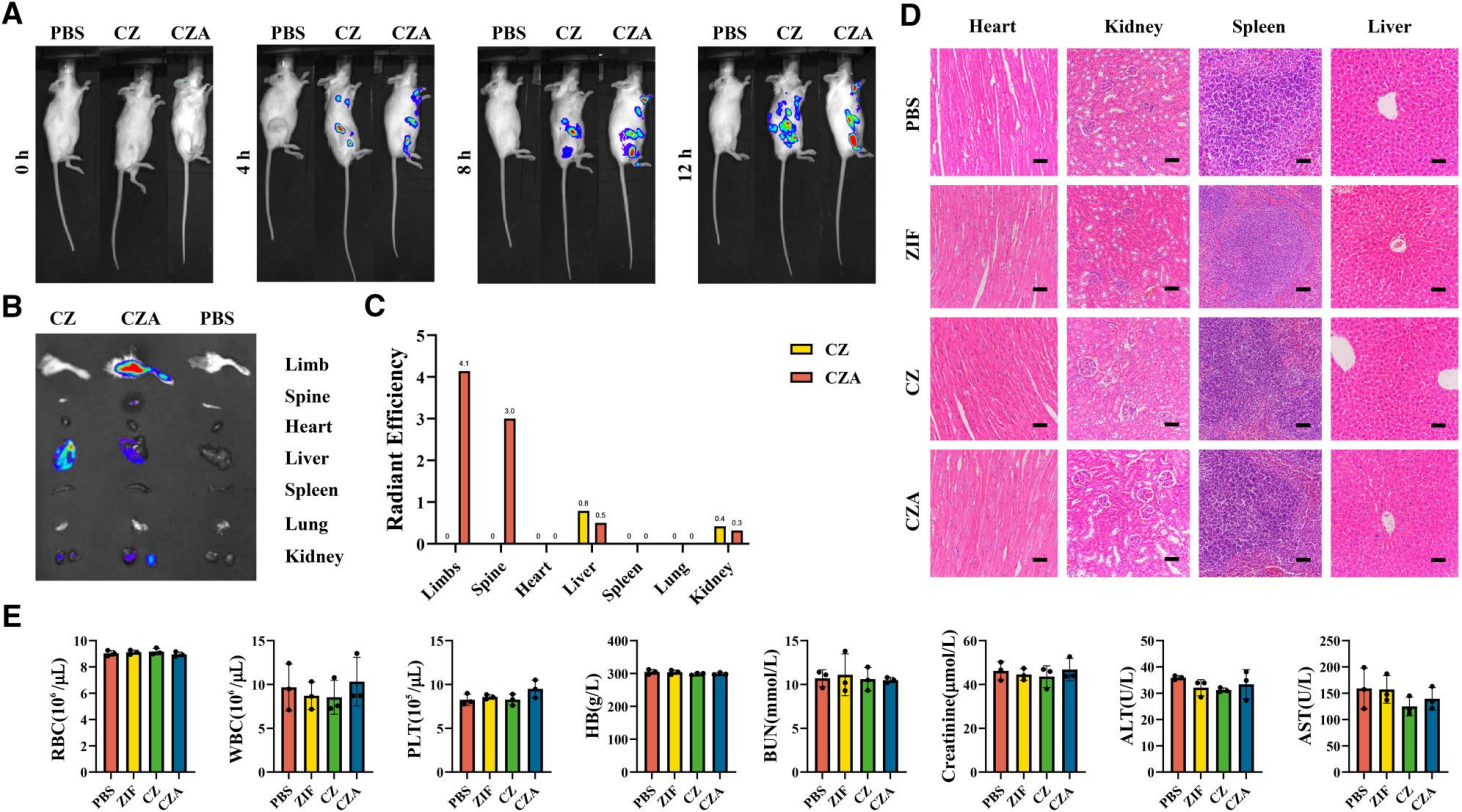
Biocompatibility and bone-targeting ability of the nanoparticles *in vivo*. (**A**) Representative whole-body IVIS images 0, 4, 8 or 12 h after the injections of PBS, CZ or CZA. (**B**) Representative IVIS images of organs 12 h after the injections of PBS, CZ or CZA. (**C**) Quantification of radiant efficiency in the organs 12 h after the injection of CZ or CZA. (**D**) Hematoxylin and eosin staining of major organs obtained from PBS, ZIF, CZ or CZA group after 7 days and three injections (scale bars, 50 µM). (**E**) Routine hepatorenal function tests of mice subjected to different treatments (*n* = 3). Cur, curcumin; CZ, Cur@ZIF; CZA, Cur@ZIF@ASP8; IVIS, *in vivo* imaging system; PBS, phosphate-buffered saline; ZIF, zeolite imidazolate framework-8.

The targeting ability of nanoparticle drug delivery systems confers a substantial advantage for therapeutic applications. Although earlier research has established the bone-targeting capabilities of ASP8 oligopeptides, the bone-targeting efficacy of CZA remains to be verified [20]. Therefore, the nanoparticles were pre-labeled with IR780 and administered to mice through tail vein injection. Fluorescence imaging was performed at 4, 8 and 12 h postinjection to assess their systemic distribution. The *in vivo* fluorescence analysis (Figure 5A) demonstrated that, by 6 h postinjection, ASP8-modified CZ nanoparticles exhibited significantly greater accumulation in the bone region compared to the control group. Images of isolated organs (Figure 5B and C) further revealed that at 12 h, the nanoparticles in the CZ group were primarily concentrated in the liver and kidneys, whereas the fluorescence signals in these two organs were significantly weaker following CZA administration. However, the CZA group presented notable fluorescence signals in the femur and spine. The results indicate that modifying nanoparticles with ASP8 can increase their accumulation in bone tissue, thus promoting the drug’s therapeutic impact on this specific tissue.

After confirming the bone-targeting properties of the nanoparticles, their biosafety and therapeutic effects were assessed *in vivo*. The nanoparticles were injected to C57/BL6J mice via the tail vein every 3 days. Notably, all the mice survived until the end of the experiment and received three injections. Following treatment, tissue samples from the heart, liver, spleen and kidneys of the mice were obtained, and HE staining demonstrated no noticeable signs of pathological alterations (Figure 5D). Finally, blood samples were collected from the mice for analysis. These results indicated the lack of significant changes in routine blood parameters or liver and kidney function-related indicators (Figure 5E). These findings demonstrate the safety of the nanoparticles during treatment. *In vivo* toxicity assessments demonstrated that the CZA nanoparticle system caused no toxic effects or side effects on the major organs of the animals.

### Therapeutic potential of the nanoparticles *in vivo*

After verifying the biocompatibility and bone-targeting potential of the nanoparticles, an OVX mouse model, which has been widely recognized in numerous studies as a reliable animal model for investigating PMOP [21, 22], was established and confirmed by DXA (Supplementary Figure S2). The nanoparticles were administered to OVX PMOP model mice through tail vein injections every three days. During the treatment period, the mice were weighed at 3-day intervals. All mice survived until the conclusion of the experiment, and their body weights remained relatively stable without significant changes (Supplementary Figure S3).

**Figure 6.**
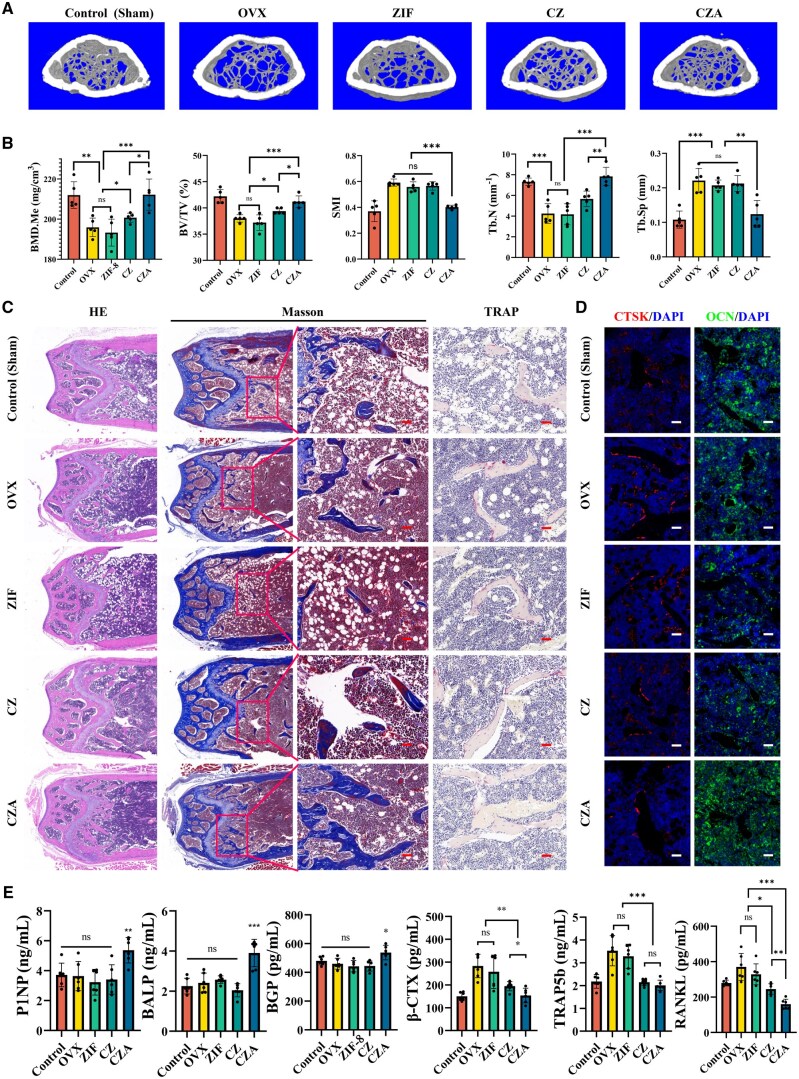
Therapeutic potential of the nanoparticles *in vivo*. (**A**) Representative 3D reconstructed microcomputed tomography (micro-CT) images of the bone microarchitecture in the distal femurs of the sham, OVX, ZIF, CZ and CZA groups. (**B**) Quantitative analysis of bone mineral density (BMD), Tb. BV/TV, SMI and Tb. N and Tb. Sp (*n* = 5) via micro-CT. (**C**) Representative images of HE, Masson’s trichrome and TRAP staining of bone sections from the distal femurs of the sham, OVX, ZIF, CZ and CZA (scale bars, 20 μm). (**D**) Representative images of OCN immunofluorescence staining (scale bars, 20 μm). (**E**) Quantitation of bone metabolism biochemical indicators, including P1NP, BALP, BGP, β-CTX, TRAP5b and RANKL, in the serum (*n* = 5) (ns: *P* > 0.05, *P < 0.05, **P < 0.01, ***P < 0.001). Cur, curcumin; CZ, Cur@ZIF; CZA, Cur@ZIF@ASP8; DAPI, 4′,6-diamidino-2-phenylindole; OVX, ovariectomized; TRAP: tartrate-resistant acid phosphatase; ZIF, zeolite imidazolate framework-8.

Micro-CT was employed to analyse the microstructure of distal femurs in mice. Sagittal images revealed that one month after intravenous administration of CZA, the mice displayed improved trabecular bone density and a higher volume of trabecular bone (Figure 6A). To provide a more detailed evaluation of the therapeutic effects associated with the four nanoparticle treatments, a quantitative analysis of micro-CT data was performed (Figure 6B). The findings revealed a significant increase in BMD within the CZA group compared to the OVX group (*P* < 0.05). Furthermore, the CZA group displayed a notably higher bone volume-to-total volume (BV/TV) ratio, suggesting that bone formation exceeded bone resorption. Additional osteoporosis-related indices, such as trabecular number (Tb.N), the structure model index (SMI) and trabecular separation (Tb.Sp), were evaluated. Osteoporotic conditions typically involve increased SMI and Tb.Sp, along with Tb.N and BMD reductions. Following CZA treatment, these parameters notably improved, reflecting enhanced bone mass and a reversal of osteoporosis in the mouse model.

HE, Masson’s trichrome and TRAP staining were utilized to evaluate osteogenic activity and osteoclast function (Figure 6C). Through HE and Masson’s trichrome staining, the structural characteristics of trabecular bone and the status of osteocytes in the distal femoral tissue were thoroughly examined. Additionally, TRAP staining demonstrated a notable reduction in osteoclast numbers in the CZA group, accompanied by a moderate decline in the CZ group. Notably, CZA effectively inhibits osteoclast differentiation *in vivo*.

Immunofluorescence staining of bone tissue was conducted (Figure 6D), revealing a substantial upregulation of OCN expression after CZA treatment. Simultaneously, CTSK expression was notably reduced in the CZA-treated group.

To evaluate bone metabolism-related indicators, ELISAs were conducted on mouse serum samples. Markers such as PINP, BALP and BGP were measured to reflect bone formation activity, whereas β-CTX, TRAP5b and RANKL were analysed as indicators of bone resorption processes [23–25]. Consequently, the CZA group showed significantly elevated levels of osteogenesis-related markers, including PINP, BALP and BGP, in comparison with the other groups. Further, all treatment groups displayed varying reductions in the β-CTX, TRAP5b and RANKL levels (Figure 6E). Thus, CZA effectively inhibited osteoclast differentiation *in vivo*.

These results indicate that CZA simultaneously inhibits osteoclastogenesis and promotes osteogenesis. Moreover, the bone-targeting potential of the nanoparticles increases the concentration of Cur in bone tissue, enhancing the efficacy of osteoporosis treatment.

## Discussion

During bone remodeling, the dynamic absorption and formation of bone tissue occur simultaneously. Osteoclasts release acids that dissolve bone minerals, facilitating bone resorption by the degradation of collagen fibers and other matrix components. This process is regulated by multiple signaling molecules, including cytokines and growth factors, which take on important roles in the interaction between osteoblasts and osteoclasts [26, 27]. Moreover, osteoblasts form new bone tissue to replace the resorbed areas, functioning under the influence of osteoclast activity [28, 29]. In recent years, researchers have paid significant attention to drugs that can bidirectionally regulate bone homeostasis by enhancing osteogenic capacity while inhibiting osteoclast activity [30, 31].

Among various potential treatments for osteoporosis, the natural polyphenol compound Cur has emerged as a potential candidate [32]. Numerous *in vitro* and *in vivo* studies have demonstrated that Cur improves bone density and enhances the mechanical strength of bones [33–35]. Cur facilitates bone formation while inhibiting bone resorption by modulating a range of proteins, enzymes and signaling pathways. Its antioxidant properties further contribute to both the prevention and treatment of osteoporosis. These effects are mediated through the regulation of key pathways and proteins, including the Akt/GSK3β signaling pathway, Wnt/β-catenin signaling pathway, insulin-like growth factor 1 (IGF-1), MMP9 and nuclear factor kappa B (NF-κB) [33, 36–40]. In addition, its antioxidant properties are largely associated with its capacity to reduce reactive oxygen species (ROS) generation while enhancing the activity of antioxidant enzymes. Studies have indicated that Cur regulates the Nrf2/ARE signaling pathway through various mechanisms, which in turn promotes the expression of antioxidant enzymes like superoxide dismutase and glutathione peroxidase. This improvement in cellular antioxidant defenses underscores Cur’s potential as a promising therapeutic option for treating osteoporosis [41–44].

A major concern regarding Cur is its poor bioavailability, which denotes the restricted absorption of Cur consumed by humans. Studies have suggested that intake of Cur alone does not produce the expected health advantages, largely because of its remarkably low concentrations in the body [45]. Furthermore, Cur is an unstable substance prone to degradation in diverse environmental conditions. This chemical instability can result in diminished activity and effectiveness during storage and application, thus constraining its use as a pharmaceutical compound [46]. Cur is also significantly limited by its poor water solubility, which hinders its incorporation into aqueous pharmaceutical formulations. This low solubility also results in reduced absorption and bioavailability in the digestive tract [32].

This study aims to address these challenges by developing CZA, an innovative nanosystem for targeted bone drug delivery. Built upon ZIF and modified with ASP8, CZA is designed to increase the bioavailability of Cur and enhance its therapeutic effectiveness in osteoporosis treatment. The efficacy of the orally administered Cur was also verified (Supplementary Figures S4 and S5). The results indicate that the efficacy of the orally administered Cur is comparable with that of CZA nanoparticles. CZA exhibited not only good biocompatibility and bone-targeting potential but was also an effective bone treatment a reduced dosage of 5 mg/kg administered every three days, which produced a therapeutic effect comparable with that of orally administered Cur of 200 mg/kg/day. This dose reduction significantly improves safety while maintaining efficacy. The advantages of the nanosystem are further amplified by the mild synthesis conditions of ZIF and acid-responsive degradation, supporting future clinical applications.

One of the innovations of this study is the novel application of ZIF nanoparticles for Cur delivery in osteoporosis treatment. ZIF provides significant advantages over these traditional nanoparticle carriers [47–50]. In comparison with other types of Cur nanodrug delivery systems, ZIF demonstrates mild synthetic conditions, high stability under normal pH and exceptional acid-responsive properties. These advantages are suitable to future large-scale preparation and clinical applications. Previous studies on Cur-loaded ZIF nanoparticles have focused on applications in wound healing, tumor therapy and antibacterial fields [51–54]. This study extends the application of Cur-loaded ZIF nanoparticles to the treatment of osteoporosis by incorporating bone-targeting modifications to the nanoparticles.

In this study, the nanoparticles were endowed with bone-targeting potential through the use of ASP8. ASP8 is recognized for its significant bone-targeting ability, primarily due to its distinct molecular structure and interactions with bone tissue. ASP8 consists of eight aspartic acid residues and contains several carboxyl groups in its structure. These carboxyl groups give the molecule a negative charge under physiological conditions, facilitating ionic interactions with hydroxyapatite, a prevalent component in bone tissue [55]. Our earlier analysis of the characteristics of these nanoparticles demonstrated that altering the ZIF organometallic framework with ASP8 reduces its zeta potential, imparting a negative charge. This negative charge improves the affinity of the nanoparticles for the bone tissue, effectively targeting bone tissue. Compared with other investigations into Cur-loaded nanoparticles for osteoporosis treatment, previous studies on bone-targeting techniques have mainly emphasized modifications involving alendronate (Aln) [49, 56]. In contrast, Aln tends to accumulate in the bones, leading to associated adverse reactions. In addition, as an osteoporosis therapeutic drug, Aln may interfere with baseline osteoporosis treatment in future applications. Research on ASP8 has significantly increased in recent years, and some studies have investigated its potential applications in bone tumors and assessed its feasibility for clinical treatment [14, 57]. This study further extends the application of the ASP8 bone-targeting strategy to ZIF for the treatment of osteoporosis, thereby broadening its scope of use. Studies have demonstrated that ASP8 exhibits a stronger affinity for highly crystallized hydroxyapatite than less crystallized forms, a property that enhances its effectiveness in targeting bone tissues [58]. This high affinity is primarily attributed to the charge characteristics of aspartic acid residues within its peptide chain, which facilitate the formation of stable binding interactions with the positive charge present on the hydroxyapatite surface. This characteristic significantly enhances selectivity for bone-targeting and reduces nonspecific adsorption to nonbone tissues. Although bisphosphonates can also bind to hydroxyapatite, Aln may lead to potential nonspecific interactions with other calcium-containing tissues, such as calcified regions in the soft tissue, thereby reducing targeting efficiency. Moreover, ASP8 exhibits excellent biocompatibility, as its decomposition products are amino acids, which are nontoxic, making it more suitable for long-term applications.

In addition, earlier studies have demonstrated that an acidic microenvironment boosts osteoclast activity, induces alterations in downstream cell signaling pathways, inhibits osteoblast proliferation and decreases osteoblast mineralization [59]. The acidic microenvironment within cancellous bone, particularly the pH-dependent activity of osteoclasts, is crucial for bone remodeling. Osteoclasts generate localized acidic niches (pH 4.5–5.0) through active H-ion secretion via V-ATPase pumps at the ruffled border, thereby facilitating hydroxyapatite dissolution and activation of proteolytic enzymes such as cathepsin [60]. In addition, chronic inflammation, frequently associated with age-related and endocrine alterations, is a characteristic of the bone microenvironment and plays a role in pH reduction [61]. The osteoclast-generated acidic niche in cancellous bone induces ZIF nanocarrier disintegration, further achieving spatiotemporally controlled curcumin release to pathological bone-resorptive sites and enhancing therapeutic efficacy while minimizing systemic toxicity. The pH-sensitive mechanism for releasing ZIF-associated drugs enables nanoparticles to utilize the anti-inflammatory properties and osteoclast inhibitory potential of Cur, subsequently aiding in the adjustment of the bone microenvironment and enhancing osteoporosis treatment. In summary, the integration of bone-targeting potential and pH-responsive drug release allows CZA nanoparticles to produce therapeutic effects comparable with those of high-dose oral Cur at significantly lower concentrations. This method promotes drug safety characteristics and minimizes possible adverse events, making CZA a strong contender for upcoming osteoporosis therapies.

Cells predominantly take in nanoparticles through clathrin-mediated endocytosis (CME) process [62]. CME can be categorized into several stages, including the invagination of the membrane, clathrin polymerization, vesicle formation and subsequent fusion of these vesicles with lysosomes or the endoplasmic reticulum [63]. Clathrin polymerization induces the bending of the membrane, leading to the formation of endocytic vesicles that encapsulate external materials. Importantly, the dimensions of the nanoparticles significantly influence the effectiveness of cellular uptake. A previous study showed that there exists an optimal size for nanoparticles that enhances uptake through CME [64]. Moreover, a study revealed that nanoparticles measuring approximately 100 nm are effective in promoting endocytosis, whereas larger smaller nanoparticles may be needed to achieve similar uptake results [65]. In this study, DLS analysis showed that CZ and CZA have diameters of 100 and 108 nm, respectively, which indicate the measurements suitable for cellular uptake. As a result, CZ and CZA can efficiently enter cells through the CME pathway.

The present results are significant for at least two major reasons. First, novel bone-targeting nanoparticles that utilize their unique bone-targeting and pH-responsive properties were synthesized to treat PMOP, demonstrating promising prospects for application. Second, the efficacy of Cur in treating osteoporosis was improved, addressing the challenge of its low bioavailability in this study.

Nonetheless, this research has three limitations. First, the therapeutic mechanisms of the Cur nanoparticles were not examined deeply. Second, the nanoparticles were administered at only one concentration, and the safety and therapeutic effects of nanoparticles in various concentrations were not examined. Finally, although the intravenous route significantly reduces the concentration and frequency of nanoparticle administration compared with oral Cur administration, it is less convenient than the oral route. In the next phase, we will investigate the detailed mechanisms by which CZA can be used to treat osteoporosis, assess the safety and therapeutic effects of CZA at various concentrations and further refine the dosage forms of CZA to facilitate oral administration or other straightforward methods of administration.

## Conclusions

CZA was prepared successfully, and bone tissue was targeted effectively. Given this characteristic, CZA facilitates the targeted delivery of Cur to bone tissue, enhancing bone quality by stimulating osteogenic differentiation and suppressing osteoclastic activity. These nanoparticles have demonstrated good biological safety, broadened the application range of ZIF nanoparticles and have broad prospects in osteoporosis treatment.

## Supplementary Material

rbaf028_Supplementary_Data

## Data Availability

The study’s primary data and results are presented in the main text and supplementary materials. Further inquiries can be directed to the corresponding author.
