# Supplementary material for: An acid-responsive bone-targeting nanoplatform loaded with curcumin balances osteogenic and osteoclastic functions
Source: Regen Biomater. 2025 May 5;12:rbaf028. doi: 10.1093/rb/rbaf028 (PMC12122077; doi:10.1093/rb/rbaf028)
Supplement: rbaf028_Supplementary_Data [file rbaf028_supplementary_data.pdf]

Supplementary Figures

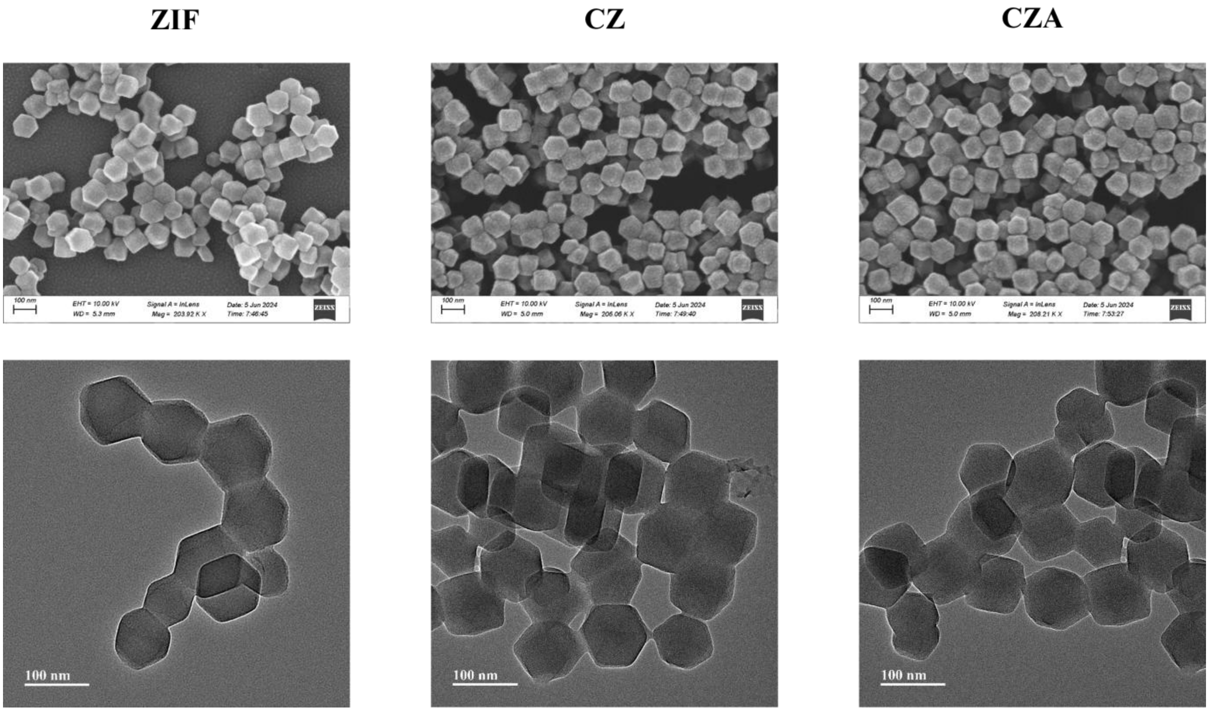

FigureS1 SEM image of ZIF, CZ and CZA

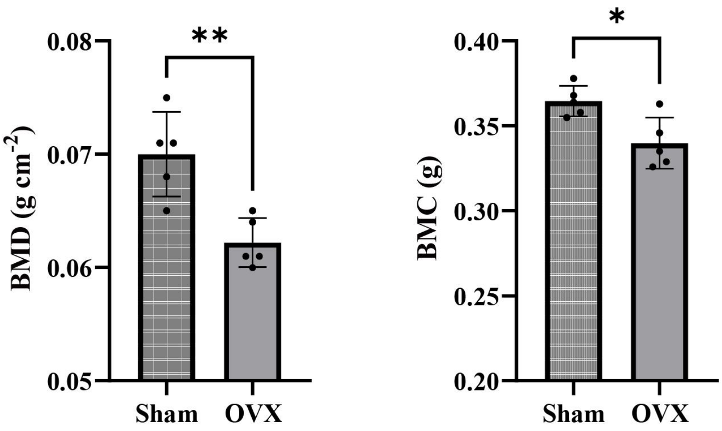

FigureS2 Results of DXA in mice one month after OVX surgery. (n = 5)

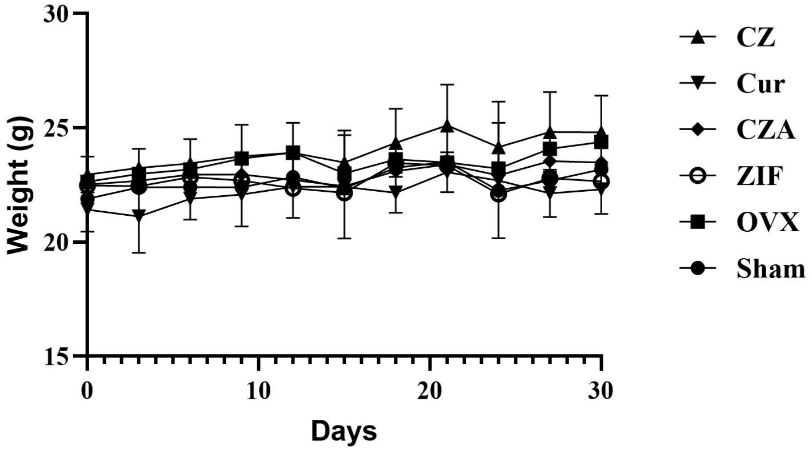

FigureS3 Weight of the mice during treatment. (n = 5)

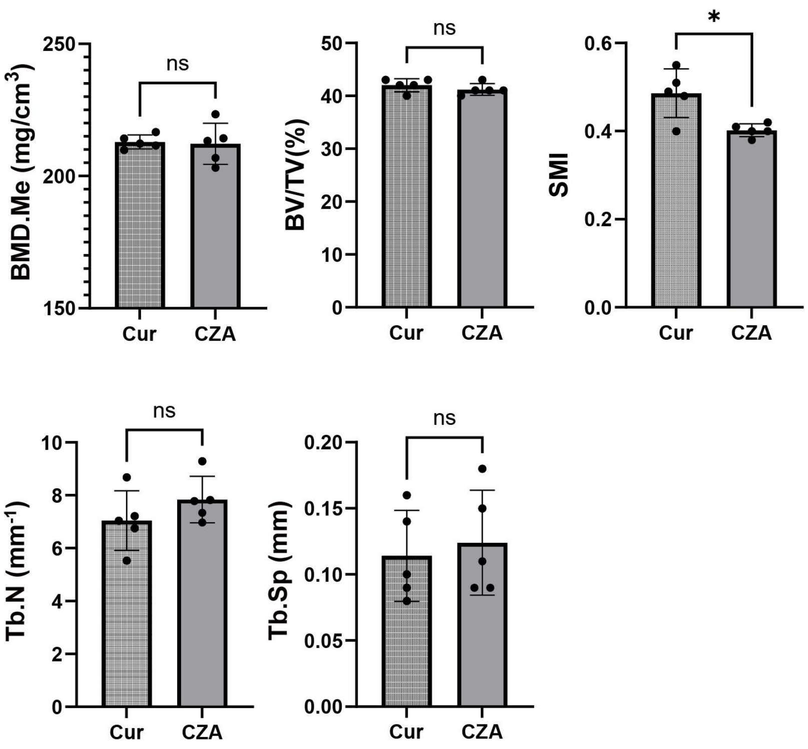

Figure S4. Quantitative analysis of the Cur and CZA groups of micro-CT for bone mineral density, Tb. BV/TV, SMI, Tb. N and Tb. Sp (n = 5) via micro-CT. (ns: P > 0.05, *P < 0.05).

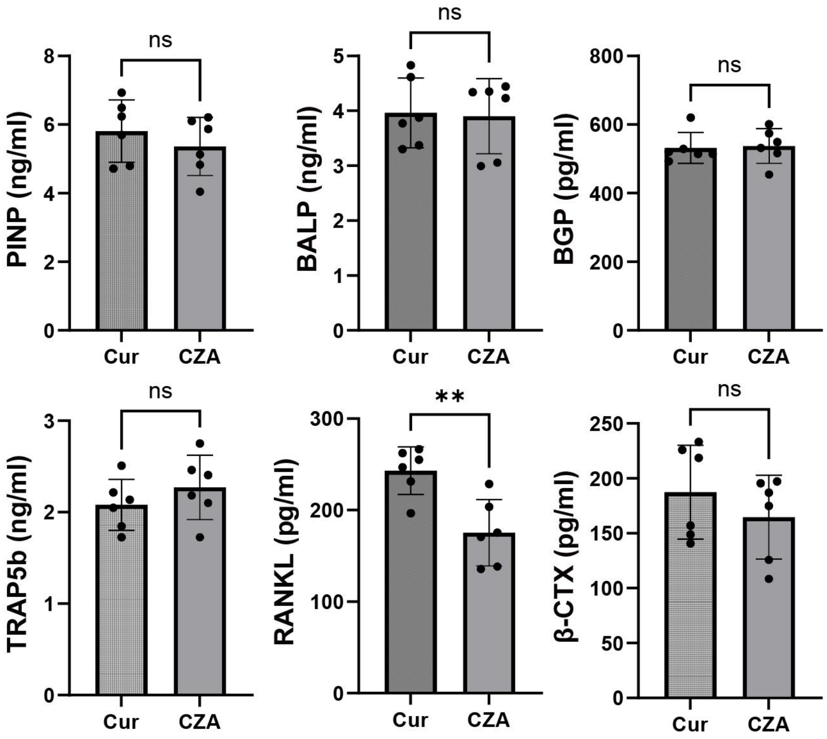

Figure S5. Quantitation of the bone metabolism biochemical indicators of the Cur and CZA groups, including P1NP, BALP, BGP, β-CTX, TRAP5b, and RANKL, in the serum (n = 5) (ns: P > 0.05, *P < 0.05,P < 0.01).**
